# An Angle Error Compensation Method Based on Harmonic Analysis for Integrated Joint Modules

**DOI:** 10.3390/s20061715

**Published:** 2020-03-19

**Authors:** Yi Hu, Yuyi Zhan, Liang Han, Penghao Hu, Bing Ye, Yue Yu

**Affiliations:** 1 School of Instrument Science and Opto-electronics Engineering, Hefei University of Technology, Hefei 230009, China; 2018170026@mail.hfut.edu.cn (Y.Z.); 18326671678@163.com (L.H.); hupenghao@hfut.edu.cn (P.H.); 2 School of Electronic Science & Applied Physics, Hefei University of Technology, Hefei 230009, China; yb0430@hfut.edu.cn; 3 R&D Department of Anhui Heli Co., Ltd, Hefei 230000, China; yuyue_sunny@126.com

**Keywords:** integrated joint module, magnetic encoder, angle error compensation, harmonic analysis

## Abstract

Nowadays, integrated joint modules are increasingly adopted in manipulators for their advantages of high integration, miniaturization and high repeatability positioning accuracy. The problem of generally low absolute positioning accuracy (namely angle measurement accuracy) must be solved before they can be introduced into the self-driven articulated arm coordinate measuring machine which is under study in our laboratory. In this study, the sources of joint module’s angle error were analyzed and the error model based on harmonic analysis was established. Two integrated joint modules were calibrated on the self-designed calibration platform and the model parameters were deduced, respectively. The angle error was then compensated in the experiments and the results demonstrated that the angle error of the joint modules was reduced by 82.03% on average. The established angle error model can be effectively applied into the self-driven articulated arm coordinated measuring machine.

## 1. Introduction

With the rapid development of industrial technology, industrial robots have gradually entered the industrial assembly line production chain and are replacing manual operations due to advantages such as speed, flexibility and low long-term investment cost. Industrial robots are widely used in automobile, mold, aerospace, shipbuilding and other mechanical processing industries; the six-axis multi-joint robot, namely the 6-DOF Manipulator, is among the most common and is used in handling, assembly, welding and surface treating processes [1]. The motion and accuracy control of the six-axis multi-joint robot have become research hotspots in this field. The manipulator motion control poses a trajectory planning problem for the entire manipulator system. Trajectory planning considers obstacle avoidance, time optimization and motion stability. Early literature has used the maximum speed or acceleration constraints to select the optimal speed or acceleration curve for determining the path and trajectory [2], the maximum principle to solve the time optimal trajectory planning [3] and various optimal algorithms of nonlinear constraint [4] and has even introduced intelligent algorithms such as neural networks, genetic algorithms or other algorithms for time optimal trajectory planning [5,6,7]. In terms of accuracy control, manipulator error can be caused by many influencing factors, further complicating the problem of error compensation. Furthermore, the serial mechanism of the manipulator enlarges the error stepwise from the base to the end, necessitating error compensation. The accuracy of the industrial manipulator can be divided into absolute positioning accuracy and repetitive positioning accuracy. Most manipulators have excellent repetitive positioning accuracy, but still currently lack absolute positioning accuracy [8]. Existing research methods for improving the manipulator absolute positioning accuracy are divided into two types: off-line and on-line error compensation [9]. On-line error compensation requires a real-time feedback device, such as a laser tracker for calibrating the kinematic parameters of the manipulator [10], to realize real-time on-line error compensation of errors caused by internal and external manipulator factors. This method is costly and requires specialized personnel to operate. Off-line error compensation does not require a real-time feedback device, has low input cost and does not rely on information from external equipment; however, it does not consider errors caused by non-geometric parameters and the calculation process is tedious [11]. Essentially, off-line error compensation uses mathematical methods to establish an error model: A calibration device composed of high accuracy reference parts is used to calibrate it, obtain an error curve, establish an error model and correct the measured value to obtain a relatively accurate value. Most of the processes involved rely only mathematical calculation. As a result of its low cost, this method is widely used in actual manipulator error compensation. Generally, the accuracy control of an industrial manipulator can identify the structural parameters by establishing a kinematic model and carrying out the forward and inverse calibrations based on the specific calibration methods for error compensation [12,13,14], or improve the positioning accuracy of a selective compliance assembly robot arm (SCARA) by compensating for the positioning error of joint angle [15].

As a result of the wide application of the manipulator, there have been problems with poor part generality caused by too many product models. It is extremely important to develop a modular manipulator with high integration, strong reliability and good versatility. Accordingly, research on modular manipulators has been conducted by Carnegie Mellon University [16], Toshiba Corporation [17], Schunk Corporation [18] and Harbin Institute of Technology Robotics Lab [19]. Later many companies have launched their integrated joint module products.

At the same time, the detection demand in the industry has derived the articulated arm coordinate measuring machine (AACMM), which has a structure similar to the manipulator. Compared with the traditional coordinate measuring machine (CMM), its high flexibility and portability gives AACMM a market advantage. Improving the measurement accuracy of this high-precision measuring equipment is particularly important. Many scholars have studied various aspects of improving the AACMM measurement accuracy. The measurement model of AACMM uses the joint angle measured by the grating sensor as a variable and includes the structural parameters of AACMM. A main research direction is improving the identification accuracy of the structural parameters of the AACMM system by studying the calibration technology [20,21]. In References [22,23], the angle error compensation was achieved by establishing the joint angle error model, thereby improving the accuracy of angle measurement. Reference [23] adopted the harmonic analysis method for modeling, and the highest order is 3. The experimental results show that these studies are helpful for improving the AACMM measurement accuracy. For the grating sensor, Reference [24] used cubic spline, polynomial and harmonic analysis methods to establish the angle error model. The comparison showed that the harmonic analysis has the best compensation effect, and the fitting function contains a wider frequency domain, which is closer to the actual error curve.

In Reference [25], we proposed a new type of self-driven AACMM that realizes automatic measurement. To miniaturize the joint with drive, we intend to introduce the integrated joint modules into the self-driven AACMM. From the robot perspective, the repetitive positioning accuracy is mainly considered in the integrated joint module development, while from the AACMM perspective, we hope it is capable of high-accuracy angle measurement. For this purpose, the angle error of the integrated joint module needs to be corrected so that it can be used in the self-driven AACMM. In this paper, the angle error model of the integrated joint module is established by harmonic analysis, and the harmonic orders are determined by the amplitude and phase of harmonic components.

## 2. Angle Error Analysis of Joint Module

The integrated joint module is composed of motor, driver, encoder and harmonic reducer. The angle encoder is at the rear end of the motor shaft to sense motor in real time. The motor shift is connected with the wave generator of the harmonic reducer. The steel wheel of the harmonic reducer is fixed, and its flexible wheel outputs low rotational speeds. In this way, an error chain is formed between the encoder and the final output via harmonic reducer, so, the angle measurement error of the joint module is not only related to the encoder itself, but also influenced by the harmonic reducer. 

### 2.1. Magnetic Encoder Angle Error

**Figure 1:**
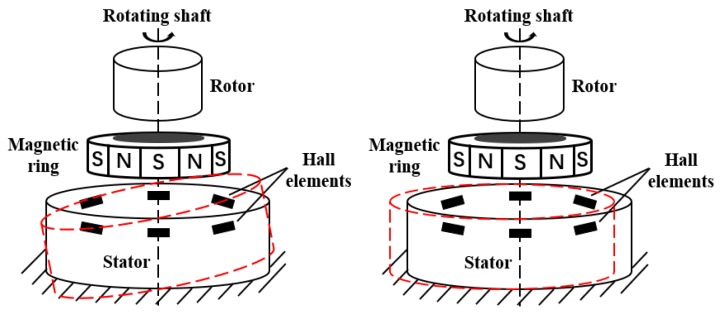
Tilt and eccentric error in manufacturing and installation.

The absolute magnetic encoder is used in the joint module. Compared with the grating encoder, the magnetic encoder has the advantages of simple structure, fast response speed, strong resistance to environmental interference, easy integration in digital circuits and wide use in speed and position detection [26]. Based on the pole pairs in the magnetic ring, magnetic encoders can be divided into single-pole and multi-pole encoders. In single-pole magnetic encoders, the magnetic ring rotates for one circle and the Hall element outputs a sinusoidal signal of one period. To obtain a higher resolution, the original signal frequency must be multiplied. By the design of different distribution of Hall sensors in single-pole magnetic encoders, the original analog signals of different initial phases can be obtained and the calibration and look-up table subdivision algorithm can be introduced to solve the angle. After the single-pole encoder resolution is improved to a certain degree by algorithm, it is difficult to improve further, but increased magnetic poles can make the resolution subdivision more detailed. Presently, there already is the design of absolute multi-pole magnetic encoder with index track and subdivision track combined [27]. For the multi-pole magnetic encoder, which needs to process continuous analog signal to achieve continuous angular output, the region must be divided and the location of the region determined according to the magnetic pole distribution. Therefore, the positioning error of pole distribution of the magnetic ring will be considered during the manufacturing process. The magnetic encoder signal is generated by the relative displacement of the Hall elements and the magnetic ring; therefore, the accuracy of the relative position of the Hall elements and the magnetic ring affects the encoder output signal accuracy. In the manufacturing and installation process of the signal generating part, tilt and eccentricity between the Hall elements and the magnetic ring (as shown in Figure 1) will inevitably introduce low-frequency error into the generated signal. Eccentricity will cause first-order harmonic error, and tilt will lead to second-order harmonic error [28]. Some subdivision error will also cause high-frequency error [29]. The error is generally periodic, therefore the harmonic analysis method should be adopted.

### 2.2. Harmonic Reducer Angle Error

**Figure 2:**
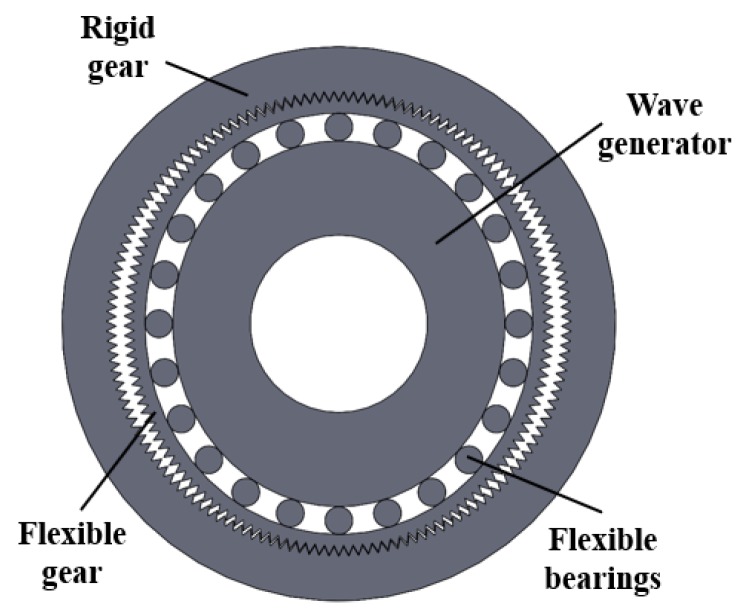
Structural schematic of harmonic reducer.

Compared with other mechanical transmissions, the harmonic reducer achieves greater transmission ratio, higher accuracy, lower noise and better working stability with fewer parts, smaller size and lighter weight [30]. The harmonic reducer generally comprises a rigid gear, flexible gear and wave generator and the transmission method of this assembly is based on the principle of elastic deformation. Figure 2 shows the reducer used in this experimental platform. One of the three basic components is generally fixed while the others are drive and driven components. When the rigid gear is fixed, the wave generator drives and the flexible gear is driven. The wave generator is installed in the flexible gear, then the wave generator cam generates a deformation wave that is transmitted to the flexible bearings and then transmitted to the flexible gear, causing deformation. With the continuous rotational deformation of the wave generator, the flexible gear will also generate periodic elastic deformation and the corresponding displacement will be generated by meshing with the rigid gear to create dynamic transmission [31].

The harmonic reducer error is classified based on its components, including the radial size error and radial run-out error of the wave generator, the geometric eccentricity error and the movement eccentricity error caused by processing and installing the rigid and flexible gears, the backlash error caused by gear backlash between the rigid and flexible gears. These transmission errors are periodic. In Reference [32], the transmission error signal of the harmonic drive was collected by a device. The signal shows that there are weak low-frequency components and strong high-frequency components in the harmonic reducer error, indicating suitability for the introduction of the harmonic analysis method.

Error compensation is divided into on-line error compensation and off-line error compensation, and the effect of on-line compensation is generally better. However, due to the integrated design of the joint module, this paper selected the harmonic analysis method based on the angle error characteristics of the joint module. Compared with on-line compensation, the cost is greatly reduced, and it is more convenient because it only relies on mathematical algorithms. The compensation process combines the amplitude and phase of the error harmonic to determine more harmonic orders, making the fitting curve much closer to the actual error.

## 3. Model of Angle Error of Joint Module

The angle error of the joint module is a function *y* = *f*(*x*) with a 2π period. If the Dirichlet condition is satisfied, any periodic signal can be expanded into Fourier series [33]. The function of the angle error is absolutely integrable over a period, is of bounded variation in any given bounded interval, and is continuous in any given bounded interval. Therefore, the angle error of the joint module satisfies the Dirichlet condition and can be expressed by Fourier series as

(1) $$\begin{array}{cc} y=f(x)\approx \varphi (x) & =A_{0}+A_{1}\cos x+A_{2}\cos2x+\cdots +A_{n}\cos nx\\ & +B_{1}\sin x+B_{2}\sin2x+\cdots +B_{n}\sin nx \end{array}$$

where

$$\begin{array}{l} A_{0}=\frac{1}{2\pi }\int_{0}^{2\pi }f(x)dx\\ A_{i}=\frac{1}{\pi }\int_{0}^{2\pi }f(x)\cos ixdx\\ B_{i}=\frac{1}{\pi }\int_{0}^{2\pi }f(x)\sin ixdx\\ i=1,\;2,\;\cdots ,\;n \end{array}$$

Equation (1) can be simplified as

(2) $$\begin{array}{c} y\approx \varphi (x)=C_{0}+\sum_{i=1}^{n}C_{i}\sin(i\theta +\varphi_{i})\\ C_{i}=\sqrt{A_{i}^{2}+B_{i}^{2}}\\ \varphi_{i}=\arctan\left(\frac{A_{i}}{B_{i}}\right) \end{array}$$

where $C_{0}=A_{0}$, $C_{i}$ is the amplitude of the *i*-th harmonic and $\varphi_{i}$ is the phase of the *i*-th harmonic. The analysis method of expanding the periodic function of error into Fourier series as described above is called harmonic analysis.

To construct a model based on the Fourier series, the highest order *n* of the expansion must first be determined. According to the Shannon sampling theorem, when the sampling point *N* is even, the highest order *n* can only be calculated to *N*/2; when *N* is odd, it can only be calculated to (*N*-1)/2 [34]. When the sampling point *N* is small due to insufficient information, harmonics higher than *N*/2 order cannot be separated but are instead mixed in harmonics lower than *N*/2 order. The mixing rules are shown below:

(3) $$\begin{array}{l} a_{i}=A_{i}+\sum_{k=1}^{\infty }\left(A_{kN-i}+A_{kN+i}\right)\\ b_{i}=B_{i}+\sum_{k=1}^{\infty }\left(B_{kN+i}-B_{kN-i}\right) \end{array}$$

where $a_{i}$ and $b_{i}$ are actual harmonic coefficients, including higher harmonic, and $A_{i}$ and $B_{i}$ are theoretical harmonic coefficients. Assuming that *N* is 37, according to the Shannon sampling theorem, the highest order *n* can be calculated to 18, and the actual harmonic coefficients after harmonic mixing can be obtained from Equation (3) as follows:

(4) $$\begin{array}{l} a_{1}=A_{1}+A_{36}+A_{38}+A_{73}+\cdots \\ b_{1}=B_{1}+B_{38}-B_{36}+B_{75}-\cdots \\ \vdots \\ a_{17}=A_{17}+A_{20}+A_{54}+A_{57}+\cdots \\ b_{17}=B_{17}+B_{54}-B_{20}+B_{91}-\cdots \\ a_{18}=A_{18}+A_{19}+A_{55}+A_{56}+\cdots \\ b_{18}=B_{18}+B_{55}-B_{19}+B_{92}-\cdots \end{array}$$

Generally, harmonic coefficients are gradually attenuated as the order increases, therefore higher order coefficients have low influences. It is apparent from Equation (4) that in low-order harmonic coefficient analysis, the influence can be ignored due to the large order span of the unseparated high-order and theoretical harmonics. As the order gradually increases, the theoretical harmonic order gradually approaches the unseparated higher order; even at the 18th order, it is directly affected by the adjacent 19th order. As such, even if the harmonic coefficient is attenuated due to increased order, the mixing of the coefficients of adjacent orders with similar magnitudes will still greatly affect the analysis results. Moreover, there may be several high orders with larger coefficients in the actual harmonic, which will seriously affect the results when they are mixed into the adjacent harmonic coefficients. There are 37 sampling points in the experiment. To reduce the influence of harmonic mixing, it is appropriate to use *n* as high as 17.

Combined with Equation (2), the angle error can be expressed as

(5) $$\begin{array}{c} \Delta \theta \approx \varepsilon (\theta )=C_{0}+\sum_{i=1}^{17}C_{i}\sin(i\theta +\varphi_{i})\\ C_{0}=\frac{1}{17}\sum_{i=1}^{17}\Delta \theta_{i} \end{array}$$

where Δθ is the angle error measured by the experiment, and ε(θ) is the angle error of harmonic fitting.

## 4. Calibration Experiment of Angle Error of Joint Module

### 4.1. Calibration Experiment and Experimental Data

**Figure 3:**
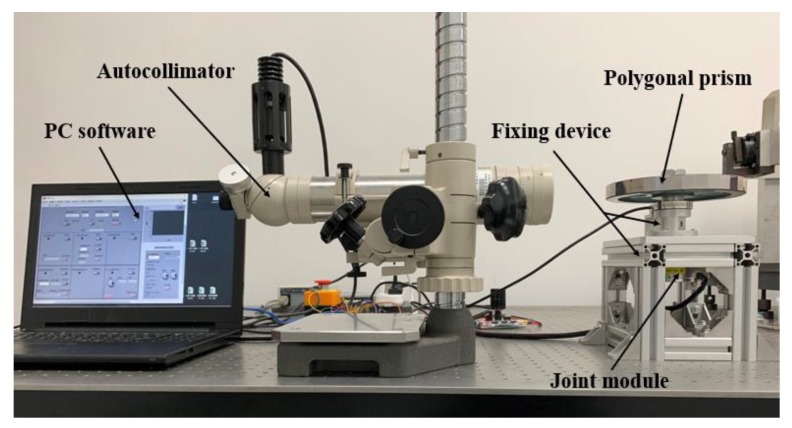
Experimental calibration platform.

To correct the joint module angle error, a joint module calibration experiment platform was designed and built using the autocollimator and a 36-sided prism, as shown in Figure 3. A fixing device was designed and manufactured to connect the joint module and the 36-sided prism. The bottom of the joint module is fixed and the output shaft is driven by the motor to rotate the prism synchronously. The prism and the autocollimator provide the angle reference for calibrating the joint module angle error.

**Figure 4:**
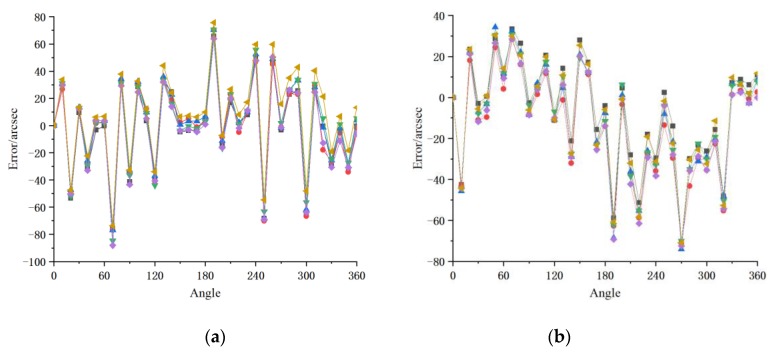
Original experiment error of joint modules: (**a**) joint module a; (**b**) joint module b.

During the experiment, the angle of the prism was first fine-tuned by making the cross image in the autocollimator eyepiece reflect back and align with the scale line in the eyepiece. In this process, the self-adjusting structure of the autocollimator can be used for adjustment. After the adjustment was completed, the position of the prism was fixed and recorded as the initial zero position; the current angle of the joint module was also recorded. Next, the PC software was used to control the joint module to rotate 10° and the position of the cross image in the eyepiece of the autocollimator was used to fine-tune the joint module angle until the cross image and the scale line coincided again. At this point, the joint module had rotated 10° and the joint module angle was read again. If the difference from the previous angle is not 10°, then the difference from 10° is the angle error. Similarly, the 36-sided prism was driven for one rotation and 37 joint module angles, corresponding to each standard 10° of the prism were obtained. The above experiments were repeated on two joint modules (named a, b) of the same type every three days within three weeks. Finally, as shown in Figure 4, 6 groups of angle data of two joint modules were obtained with 37 equal points in each group. It is apparent that the trend of angle error is basically the same with good repeatability, indicating suitability of error compensation. It also shows that the high frequency component of the angle error is larger than that of the general circular grating sensor.

### 4.2. Error Compensation and Results

**Figure 5:**
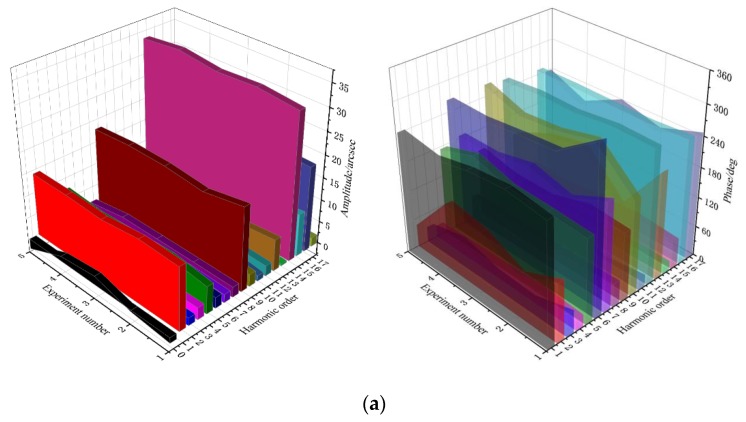

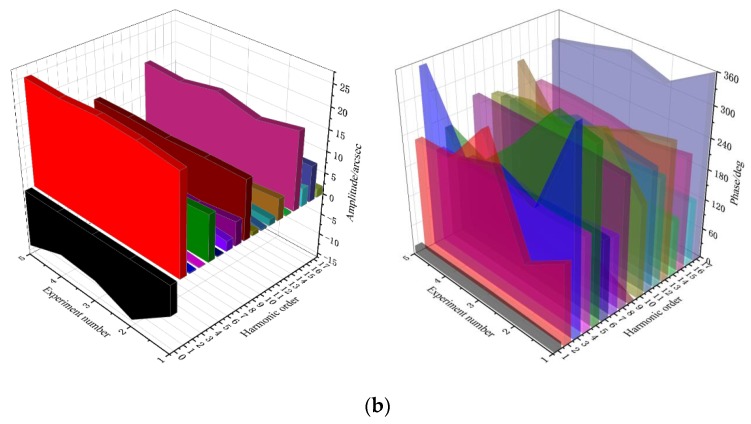
Harmonic characteristic diagram of 5 times the angle error of joint modules: (**a**) joint module a; (**b**) joint module b.

For the 6 groups of measured data, the first 5 groups were used to establish the error compensation model while the last group was used to verify the compensation model effect. First, for each group of measurement data, $A_{i}$ and $B_{i}$ in Equation (1) were solved by the least square method to obtain the harmonic amplitude $C_{i}$ and phase $\varphi_{i}$ of each order of each group, as shown in Figure 5.

**Figure 6:**
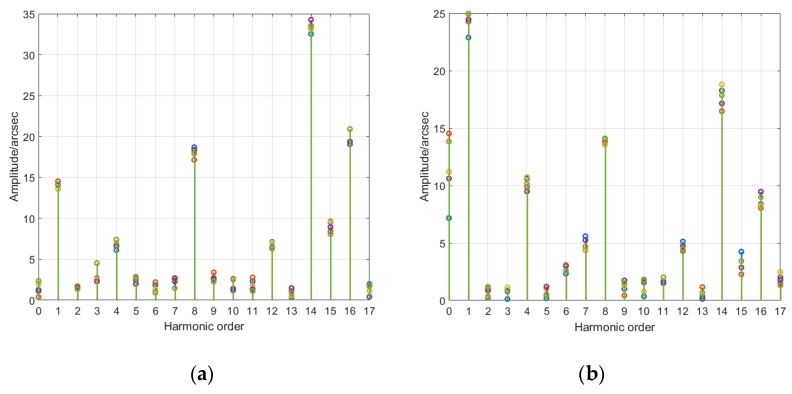
Harmonic amplitude-order diagram of joint module angle errors: (**a**) joint module a; (**b**) joint module b.

To fit an optimal harmonic error model in the actual fitting process while avoiding extra error caused by over fitting, only the harmonic components of finite order with prominent amplitude are considered, rather than harmonic components of all orders [35]. The amplitude-order diagram in Figure 6 directly shows that the harmonic orders having greater impacts on angle error in joint module a are orders 1, 8, 14 and 16. The harmonic orders having greater impacts on angle error in joint module b are orders 0, 1, 4, 8, 14, 16. Some controversial orders must be judged in conjunction with subsequent phase changes. 

**Figure 7:**
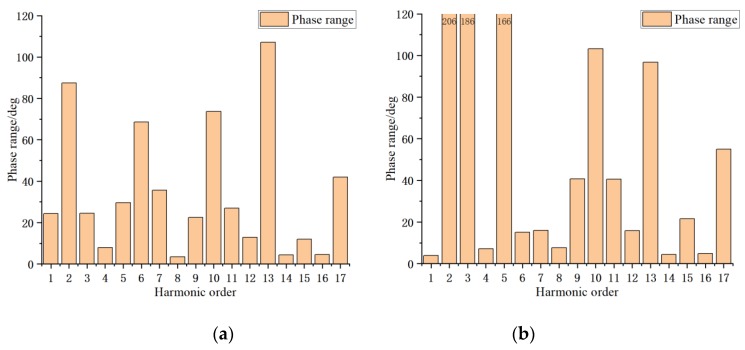
Phase range of harmonics in each order of angle error of joint modules: (**a**) joint module a; (**b**) joint module b.

Theoretically, system errors such as eccentric error generally do not change with time and neither do the amplitudes and phases of the corresponding harmonic orders that they cause [36]. Therefore, the order can be determined by combining the phase variation of each order in each group of experiments (see Figure 7). Among them, the phase variations of orders 4, 8, 12, 14, 15 and 16 of joint module a are small at approximately 10°. Combined with orders of large amplitude, it is finally determined that only orders 1, 4, 8, 12, 14, 15, 16 are retained in the error model. The phase variations of orders 1, 4, 6, 7, 8, 12, 14 and 16 of joint module b are also small; combined with orders of large amplitude, it is finally determined that orders 0, 1, 4, 7, 8, 12, 14, 16 are retained in the error model. Therefore, the harmonic expressions of the angle errors of joint modules a and b can be expressed as

(6) $$\begin{array}{cc} \varepsilon_{a}(\theta )= & C_{1}\sin(\theta +\varphi_{1})+C_{4}\sin(4\theta +\varphi_{4})+C_{8}\sin(8\theta +\varphi_{8})\\ & +C_{12}\sin(12\theta +\varphi_{12})+C_{14}\sin(14\theta +\varphi_{14})\\ & +C_{15}\sin(15\theta +\varphi_{15})+C_{16}\sin(16\theta +\varphi_{16}) \end{array}$$

(7) $$\begin{array}{cc} \varepsilon_{b}(\theta )= & C_{0}+C_{1}\sin(\theta +\varphi_{1})+C_{4}\sin(4\theta +\varphi_{4})+C_{7}\sin(7\theta +\varphi_{7})\\ & +C_{8}\sin(8\theta +\varphi_{8})+C_{12}\sin(12\theta +\varphi_{12})\\ & +C_{14}\sin(14\theta +\varphi_{14})+C_{16}\sin(16\theta +\varphi_{16}) \end{array}$$

To reduce the impact of accidental error, after averaging the amplitude and phase of five groups of error data, the final error compensation models are as follows

(8) $$\begin{array}{cc} \varepsilon_{a}(\theta )= & 14.10\sin(\theta +243.75)+6.91\sin(4\theta +26.73)+18.05\sin(8\theta +46.50)\\ & +6.66\sin(12\theta +264.24)+33.36\sin(14\theta +20.82)\\ & +8.94\sin(15\theta +57.88)+19.60\sin(16\theta +247.91) \end{array}$$

(9) $$\begin{array}{cc} \varepsilon_{b}(\theta )= & -11.47+24.31\sin(\theta +15.81)+10.18\sin(4\theta +187.75)\\ & +4.86\sin(7\theta +147.06)+13.85\sin(8\theta +258.45)\\ & +4.68\sin(12\theta +213.24)+17.71\sin(14\theta +115.24)\\ & +8.64\sin(16\theta +128.30) \end{array}$$

**Figure 8:**
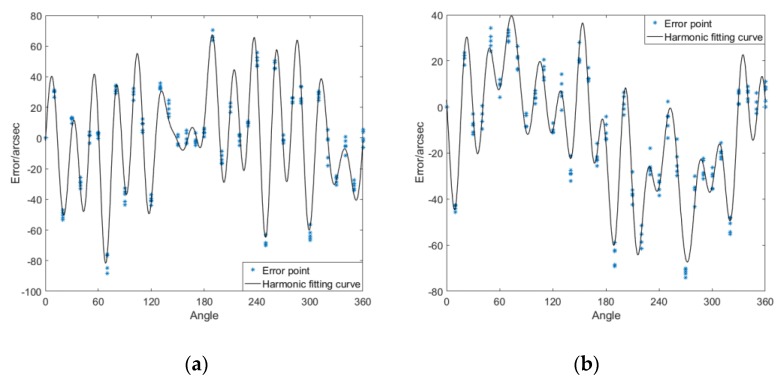
Curve of the angle error model and error scatter diagram: (**a**) joint module a; (**b**) joint module b.

**Figure 9:**
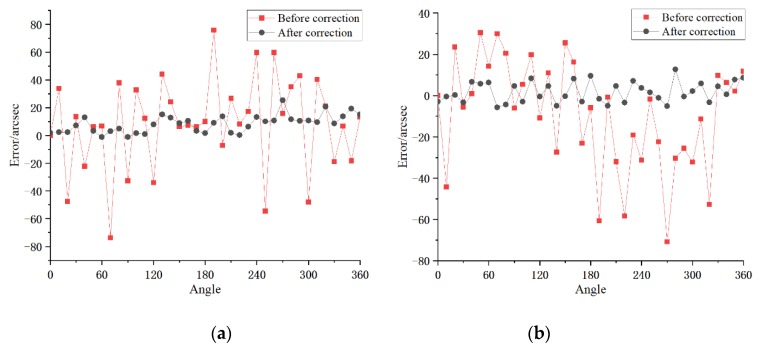
Before and after comparison of angle error compensation: (**a**) joint module a; (**b**) joint module b.

The curve of the error model is shown in Figure 8. The error model is applied to the 6th group of data, which did not participate in the modeling, and the effect of error compensation is shown in Figure 9.

Before the compensation, the peak values of error of joint modules a and b were 149.53 and 101.35 arcsec, respectively. After applying the error model compensation, any joint module angle error between 0–360° can be corrected. The peak values were reduced by 82.20% and 81.85%, to 26.62 and 18.40 arcsec, respectively. The angle measurement accuracy was significantly improved for the joint module whose internal structure was a magnetic encoder with limited precision. 

## 5. Conclusions

In this paper, the integrated joint modules in the robot are used as the driving joints of the self-driven AACMM and the angle errors of the joint modules are corrected by establishing the angle error model. The angle measurement error of the joint module is not only related to the encoder itself, but also influenced by the harmonic reducer. The high frequency component of the angle error is larger than that of the general circular grating sensor. The established error compensation model uses the method of eliminating the weak harmonic orders and retaining the strong harmonic orders in the 17 harmonic components, rather than, simply obtaining the first three harmonic orders, to make the fitting curve much closer to the actual error. The fixing mechanism for the calibration experiment was designed and manufactured. The mechanism is easy to install and its coaxiality is adjustable. The experimental data used for modeling were obtained and the angle error was reduced by 82.03% on average after compensation using the error model. This research will be helpful to improve the measurement accuracy of the self-driven AACMM under study and would be helpful to improve the absolute positioning accuracy of robot.
